# Electrospun core–sheath PCL nanofibers loaded with nHA and simvastatin and their potential bone regeneration applications

**DOI:** 10.3389/fbioe.2023.1205252

**Published:** 2023-07-26

**Authors:** Chenghui Qian, Yubo Liu, Si Chen, Chenyang Zhang, Xiaohong Chen, Yuehua Liu, Ping Liu

**Affiliations:** ^1^ Shanghai Stomatological Hospital and School of Stomatology, Fudan University, Shanghai, China.; ^2^ Shanghai Key Laboratory of Craniomaxillofacial Development and Diseases, Fudan University, Shanghai, China; ^3^ School of Materials and Chemistry, University of Shanghai for Science and Technology, Shanghai, China; ^4^ Shanghai Engineering Technology Research Center for High-Performance Medical Device Materials, Shanghai, China

**Keywords:** core-sheath structure, nanofibers, sustained release, coaxial electrospinning, simvastatin

## Abstract

**Introduction:** Drugs and biocompatible nanoparticles have raised significant potential in advancing the bone regeneration. Electrospinning technology enables the full realization of the value of drugs and nanoparticles.

**Methods:** In this study, we have successfully fabricated core–sheath nanofibers solely composed of polycaprolactone (PCL) polymer. Simvastatin (SIM) was confined to the core of the nanofibers while nanohydroxyapatite (nHA) was loaded on the nanofiber surface.

**Results:** All the prepared nanofibers exhibited a cylindrical micromorphology, and the core–sheath structure was exploited using a Transmission Electron Microscope. X-ray pattern results indicated that SIM was in an amorphous state within nanofibers, while Fourier Transform InfraRed spectroscopy showed excellent chemical compatibility among SIM, nHA, and PCL. The actual loading of nHA within the nanofiber was determined by a thermogravimetric test due to the high melting point of nHA. Core–sheath nanofibers could release SIM for 672 h, which was attributed to the core–sheath structure. Furthermore, nanofibers loaded with SIM or nHA had a positive impact on cell proliferation, with the core–sheath nanofibers displaying the most favorable cell proliferation behavior.

**Discussion:** Such a synergistic facilitation strategy based on materials and nanostructure may encourage researchers to exploit new biomedical materials in future.

## 1 Introduction

Bone defects caused by external environmental stimuli remain a rapidly increasing trend, and the national healthcare expenditure is anticipated to substantially increase in the foreseeable future (Migliorini et al., 2021; Tan et al., 2021; Zhang et al., 2021). Autologous or allogeneic bone transplantation is a conventional method for treating bone defects (Smrke et al., 2007). However, the risks of cross-infection, immune rejection, and inadequate donor sources pose significant challenges to the treatment process of bone defects (Wang and Yeung, 2017). Fortunately, biocompatible materials that stimulate bone regeneration (such as drugs, nanoparticles, etc.), can accelerate the process. Recently, a variety of drug-loading methods have been utilized to administer drugs for bone regeneration, including 3D printing (Fitzpatrick et al., 2021; Wang et al., 2021; Ayran et al., 2022), nanoparticles (Pouroutzidou et al., 2021), hydrogels (Hajiabbas et al., 2020; Yu et al., 2022), and electrospinning (Ferreira et al., 2021; Tabia et al., 2021; Toprak et al., 2021).

Electrospinning is a prevalent technology for top-down production of nanofibers, which utilizes high-voltage electrostatics to draw working fluids into filaments (Rezaei et al., 2022; Yin et al., 2022; Basar et al., 2023; Boaretti et al., 2023; Zhou et al., 2023). During the electrospinning process, drugs or other nanoparticles can be effortlessly loaded into nanofibers without altering their original properties (Yin et al., 2021; Izgis et al., 2022; Pouroutzidou et al., 2022; Figueroa-Lopez et al., 2023). Traditional electrospun nanofibers with a single layer usually result in an initial burst of release. This uncontrolled drug release behavior significantly impacts the efficacy of the drug used in the throughout treatment process. Fortunately, the rapid development of advanced electrospinning technology has enabled the manipulation of nanofiber structure, which can control drug release behavior (Su et al., 2022; Teno et al., 2022; Wang et al., 2022c; Liu et al., 2023). In drug release delivery, nanostructures can directly influence drug release behavior by increasing drug storage space. Liu’s group has fabricated core–sheath nanofibers for sustained release of poorly soluble curcumin. The strategy of core–sheath nanofibers extends drug release time from 4 h to 36 h (Liu et al., 2022). Mohammad’s group fabricated the rosuvastatin-loaded polyvinyl alcohol/silk core–sheath nanofibers, which decreased the immediate release of rosuvastatin and enhanced the sustainable drug release behavior (Kalani et al., 2019). Reise’s group has prepared the Poly (L-lactide-co-D,L-lactide) core–sheath nanofibers to delay the metronidazole release, which had a better antibacterial effect than monolithic nanofibers (Reise et al., 2023).

The sustained and efficient release of drugs is largely dependent on the properties of materials involved (Heydari Foroushani et al., 2022). Materials with hydrophobic properties are capable of resisting water molecules, thus facilitating a sustained drug release behavior by prolonging the release of the drug from dosages. Several studies have demonstrated the efficacy of hydrophobic materials such as ethyl cellulose, polycaprolactone (PCL), poly (lactic-co-glycolic acid), and polylactic acid in sustaining drug release (Sánchez-Romate et al., 2020; Ahmadi et al., 2022; Wang S. et al., 2022; Dodda et al., 2022; Lee et al., 2022; Vlachopoulos et al., 2022). PCL, a hydrophobic material processing good biocompatibility, is extensively involved in the preparation of biomedical materials (Jiménez-Suárez et al., 2020; Bazzolo et al., 2021; Sánchez-Romate et al., 2021; Baghali et al., 2022; Setia Budi et al., 2022; Shahverdi et al., 2022). In the treatment of osteoporosis, simvastatin (SIM) has been exploited for inducing bone regeneration (Castro et al., 2018; Rezk et al., 2020; Karanikola et al., 2022). In addition, bioactive nanoparticles have been found to promote bone regeneration. Nano hydroxyapatite (nHA), a biological activity inorganic component of human and animal bones, has been shown to promote the repair of defective tissues (Bian and Xing, 2022; Wang Y. et al., 2022; Zhou et al., 2022).

In this work, we have adeptly fabricated core–sheath nanofibers utilizing PCL as the exclusive polymeric material. SIM, an active pharmaceutical ingredient, has been incorporated in the core layer, while the sheath layer contains nHA. The detailed characterization and description of nanofibers are elaborated upon in this work.

## 2 Materials and methods

### 2.1 Materials

White PCL particles with the molecular weight of 80,000 g/mol was supplied by Sigma (Shanghai, China). Phosphate Buffered Saline (PBS), acetone, and hexafluoro-isopropanol (HFIP) was purchased from China National Medicines Corporation Ltd., (Shanghai, China). SIM was purchased from Aladdin (Shanghai, China). nHA was purchased from McLean (Shanghai, China). BS350A CCK-8 solution was supplied from Labgic Technology Co., Ltd (Beijing, China), Primary human bone marrow derived mesenchymal stem cells (hBMSCs) and fetal bovine serum (FBS) were supplied from Sciencell (San Diego, CA, United States), alpha minimum essential medium (α-MEM) and antibiotics were obtained from Thermo Fisher Scientific (Rockford, IL, United States).

### 2.2 Electrospinning

Based on the only solvent HFIP, four polymeric solutions were prepared for single and coaxial electrospinning. 10 g PCL was used as the main polymeric, 0.5 g nHA and SIM were added into the polymeric solution to prepared suspensions and drug solutions. In coaxial electrospinning process, sheath and core solutions were prepared separately. The sheath solution was prepared by dissolving 7 g PCL and 0.5 g nHA in 100 mL HFIP, while the core solution was fabricated by dissolving 10 g PCL and SIM in 100 mL HFIP (Murali et al., 2020). Solutions containing nHA were suspended, while the solutions containing SIM were clear and transparent. The details of the four nanofibers are summarized in Table 1.

**TABLE 1:** Implementation details for fabricated nanofibers.

No	Process	Working fluid	Flow rate (mL/h)	Voltage (kV)	Average diameter (nm)
F1	Single	PCL	1	6	890 ± 160
F2	Single	PCL + SIM	1	6	900 ± 190
F3	Single	PCL + nHA	1	6	870 ± 220
F4	Coaxial	Sheath: PCL + nHA; Core: PCL + SIM	1.5; 1	6.5	1,080 ± 490

The aforementioned solutions were filled into 20 mL syringes via either 18 G needles or a coaxial spinneret. The spinneret was subsequently connected to high-voltage devices, operating within the range of 6–6.5 kV, by means of a clip. Then, the pumps were pushed to provide a constant rate for the syringes using a YFSP-T instrument (Tianjin, China). The nanofibers were collected using a drum collection device positioned at 16 cm from the spinneret. The flow rate of the three monolithic nanofibers was 1 mL/h; while for coaxial electrospinning, the flow rate of core and sheath was 1 mL/h and 1.5 mL/h. Then, nanofibers were placed in fume hood for 2 days to remove any residual solvent before being used for cell culture.

### 2.3 Characterization

#### 2.3.1 Morphology studies

The monolithic and coaxial nanofibers were subjected to spray aurum for 100 s in the presence of protective inert gas, to impart them with electrical conductivity. Thereafter, the micromorphology of these nanofibers was studied utilizing a scanning electron microscope (SEM; FEI Quanta 450 FEG, United States). The average diameter of nanofibers was deter-mined by analyzing SEM pictures at least 70 individual nanofibers (NIH ImageJ, Nation-al Institutes of Health, MD, United States). The internal structure of fibers was confirmed by utilizing a Transmission electron microscope (TEM; Hitachi, Japan).

#### 2.3.2 Physical forms and compatibility analysis

Physical forms of fibers were analyzed utilizing a Bruker–AXS X-ray diffractometer (Karlsruhe, Germany). It was carried out at 40 kV voltage and 30 mA current when the 2θ range was 10°–70°. Chemical compatibility between fiber components was analyzed by infrared Spectrometer (PerkinElmer, Billerica, United States). Fibers were directly placed on the stage and covered with a cover for observation at the range of 500–4,000 cm^–1^.

#### 2.3.3 Thermogravimetric analysis (TGA)

Accurately weighed 10 mg of samples were placed into an alumina crucible, and the TGA test was conducted by PerkinElmer Pyris 1 TGA analyzer (Boston, MA, American) from 30°C to 600°C at the certain rate of 5°C min^−1^. In addition, the test was kept in an inert gas atmosphere.

#### 2.3.4 *In vitro* drug release test and drug loading efficiency

The SIM-loaded nanofibers were determined by PBS (pH 7.2, 37.5°C) as the release medium. 10.0 mg of nanofibers was placed into a plastic tube containing 45 mL PBS and kept in a shaking incubator (37.5°C, 100 rpm, Zhejiang, China). At specific intervals, precisely determined periods of time, 4 mL of PBS solution was extracted from the tube. Following this, an equivalent volume of fresh PBS was added. The experiment was performed in triplicate. The actual amount of SIM released was calculated at *λ*_max_ = 238 nm utilizing UV-vis spectrophotometer (UV-2102PC, Shanghai, China). The drug loading efficiency (D) was measured using Eq. 1.

$$D\;\left(\%\right)=\frac{Actual\;SIM\;amount}{Theoretical\;SIM\;amount}*100\%$$ (1)

Nanofibers (10 mg) were completely dissolved in 4 mL acetone solvent for 2 h, and then diluted by PBS in 45 mL to obtain the absorbance of SIM at *λ*_max_ = 238 nm. Three parallel experiments were carried out. The actual SIM amount was calculated by the linear regression equation (*y* = 0.03865 *x* + 0.08853, *R* ^2^ = 0.999).

The first-order (F) and zero-order (Z) kinetic models are conducted to analyze the relationship between the drug release rate and time. The Higuchi (*H*) model is employed to determine whether the drug release followed dissolution release or diffusion release. And the Rigter–Peppas (*P*) model is utilized to investigate the release mechanism.

$$F=F_{0}\;\left(1-\exp\left(-k_{1}\;t\right)\right)$$ (2)

$$Z=Z_{0}+k_{0}\;t$$ (3)

$$H=k_{H}\;\left(t^{1/2}\right)+H_{0}$$ (4)

$$P=k_{P}\;\left(t^{n}\right)$$ (5)

Where *F*_*0*_, *Z*_*0*_, and *H*_*0*_ indicate the initial drug amount, *k* is the kinetic constant (including *k*_*1*_, *k*_*0*_, *k*_*H*_, and *k*_*P*_), *t* is the release time, and *n* is the drug diffusion coefficient.

#### 2.3.5 *In vitro* cell proliferation

hBMSCs between three and five passages were used for the *in vitro* experiments. hBMSCs were cultured in α-MEM containing 1% (v/v) antibiotics and 10% (v/v) FBS at 37°C in an incubator (5% CO_2_, Thermo Fisher Scientific, Rockford, IL, United States).

The cell proliferation and biocompatibility of PCL scaffolds were analyzed. The PCL membrane was cropped into a 7 mm circular shape and put in a 24-well cell plate. After ultraviolet disinfection for 30 min, the PCL membrane was fixed with a sterile steel column. hBMSCs at a density of 1*10^4^ per well were seeded. The culture medium was aspirated after incubation for 1 and 3 days, and cells were washed thrice with PBS. Plates were incubated at 37°C for 2 h after being supplemented with CCK-8 solution (100 μL each well). The absorbance of each well was determined by employing a Synergy HT microplate reader (450 nm, BioTek, Winooski, VT, United States).

#### 2.3.6 Statistical analysis

Data were conducted at least thrice and processed as mean ± standard deviation. The One-way ANOVA Tukey’s test was performed on cell proliferation data using origin 2017 software, and *p* < 0.05 means three is a significant difference between samples.

## 3 Results and discussion

### 3.1 Electrospinning

**FIGURE 1:**
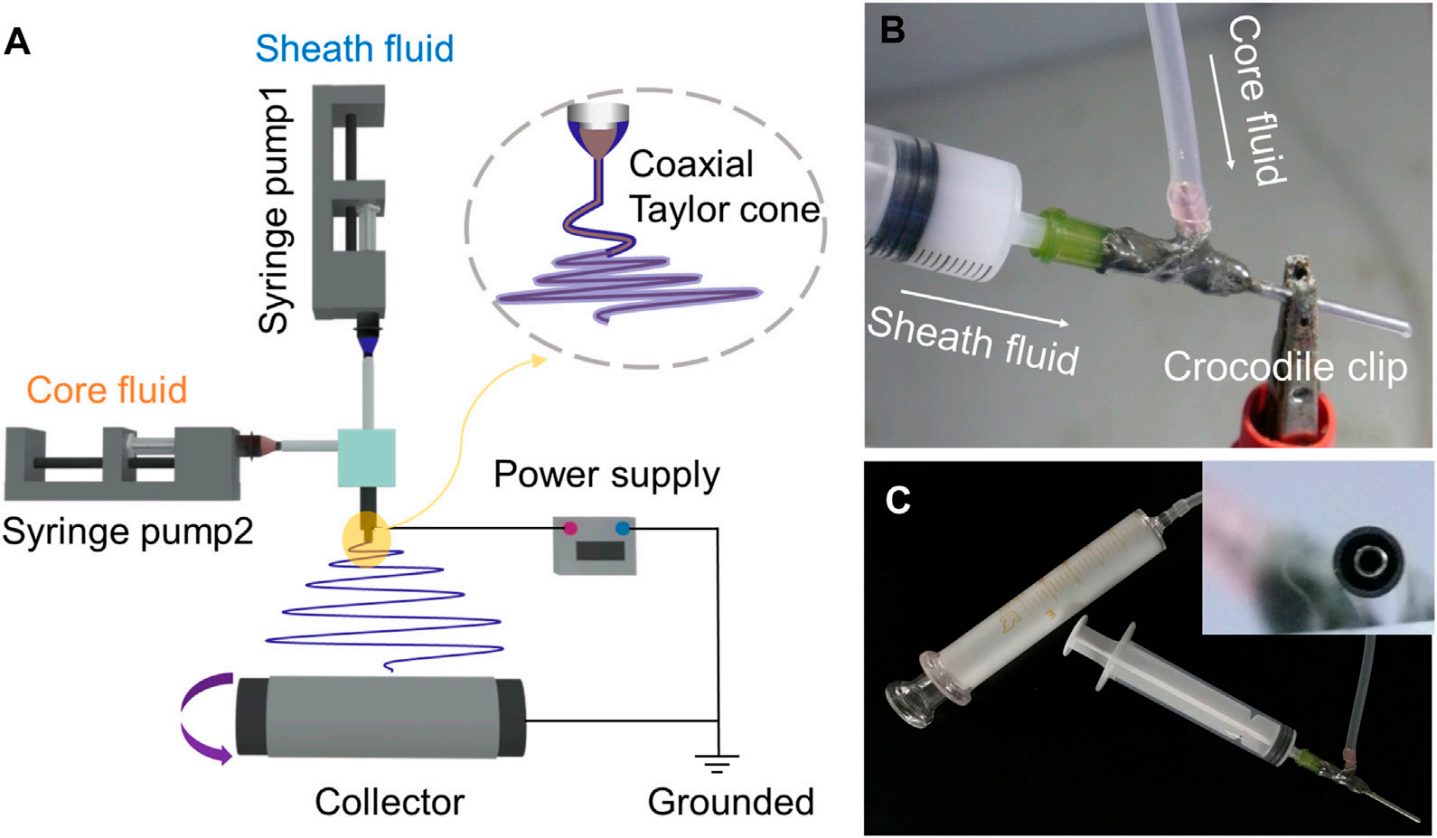
Schematic diagram of coaxial electrospinning process **(A)**. Connection between syringe and spinneret **(B,C)**.

Figure 1A shows the four components required for a successful electrospinning process, namely, syringe pump, collector, spinneret, and power supply. Figure 1B demonstrates the connection of the core fluid to the spinneret via a transparent silicone tube, while the sheath fluid is directly connected to the spinneret. The conductive clip is attached to the outer part of the coaxial spinneret to provide high voltage. Figure 1C shows the complete connection between syringe and spinneret. The bandwidth of the nanofiber membrane was exhibited in Supplementary Figure S1 in the Supplementary Materials. The incorporation of SIM or nHA in the nanofibers affected the bandwidth of the nanofiber membrane. Upon the loading of SIM into PCL nanofibers, the bandwidth of the nanofiber membrane was decreased from 8.1 cm to 6.3 cm. The addition of nHA had minimal effect on the bandwidth. However, the bandwidth of the F4 nanofiber membrane was almost halved (from 8.1 cm to 4.6 cm) during the coaxial electrospinning process. This phenomenon was not only due to the incorporation of SIM and nHA, but also related to the impact of sheath solution. The apparent change in the bandwidth of the nanofiber membrane suggests the formation of different structures.

### 3.2 Micromorphology and internal structure of prepared nanofibers

**FIGURE 2:**
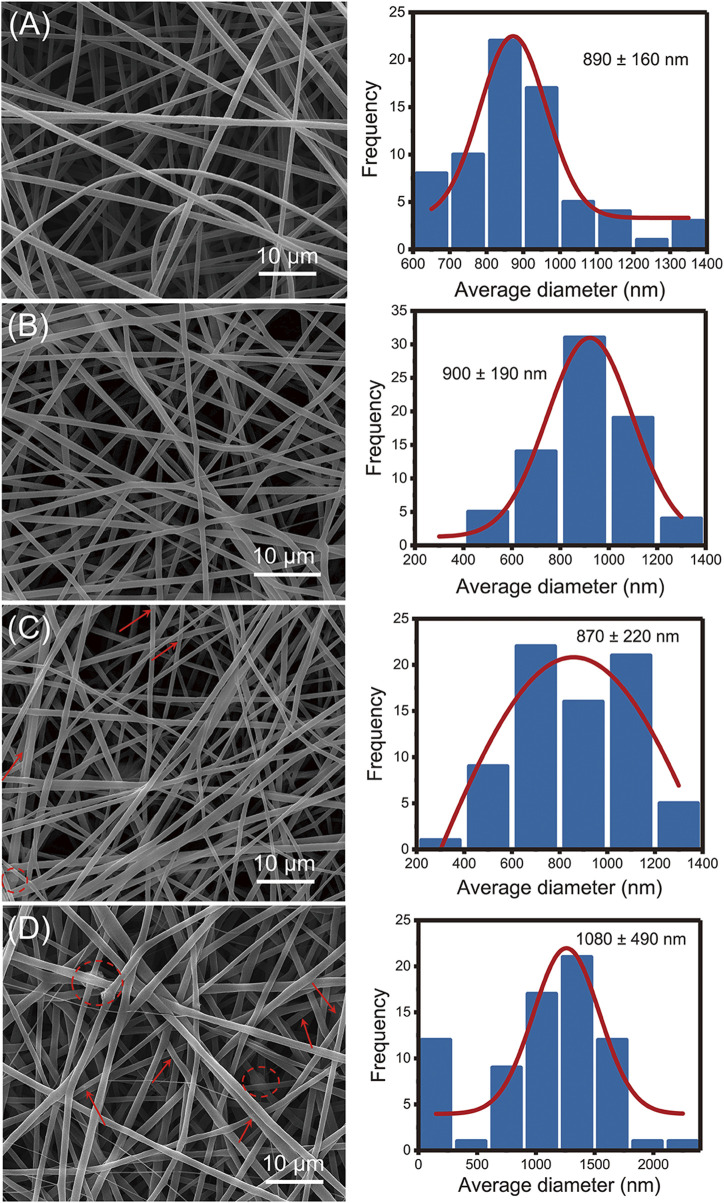
SEM images of the fabricated nanofibers: **(A)** F1 fibers, **(B)** F2 fibers, **(C)** F3 fibers, **(D)** F4 fibers.

Figure 2 showed that all prepared nanofibers had linear and cylindrical morphology. Compared with 890 ± 160 nm of F1 nanofibers, the incorporation of SIM had negligible impact on the average diameter of monolithic nanofibers (900 ± 190 nm) in Figures 2A, B. The incorporation of nHA made the nanofiber diameter more dispersed in F3 nanofibers (870 ± 220 nm). In F4 nanofibers, the average diameter was increased (1,080 ± 490 nm) compared to F1–F3 nanofibers, suggesting the presence of a core-sheath structure. As shown in Figures 2C, D, the presence of particles and agglomerated substances can be observed in F3 and F4 fibers, indicated by the red arrows and red circles, respectively. These particle materials attached to the fiber surface, suggesting the successful incorporation of nHA.

**FIGURE 3:**
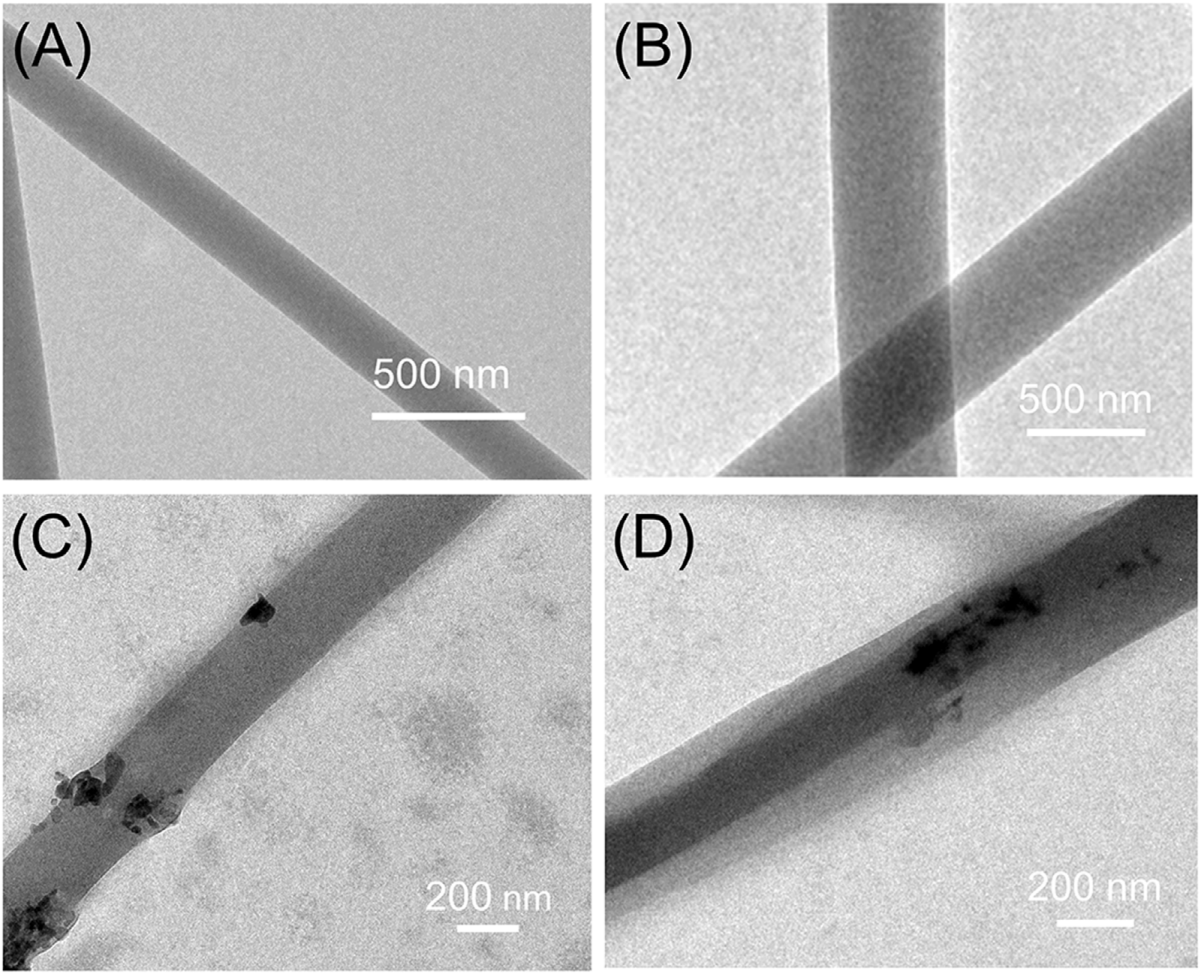
TEM images of the prepared nanofibers: **(A)** F1 fibers, **(B)** F2 fibers, **(C)** F3 fibers, **(D)** F4 fibers.

Figure 3 exhibited the internal structure of nanofibers. Based on the top-down observation, different structures will show different gray levels in TEM images. In Figures 3A–C, there was no grayscale changes in the color of the nanofibers which indicated that F1–F3 has monolithic structure. In Figure 3D, the two grayscales appeared indicated that F4 nanofibers had a clear double-layer structure. The overlapping of the gray levels of the sheath and core layers results in this clear core–sheath structure. In addition, the black particles on the surface or inside of the F3 and F4 nanofibers could be considered as nHA.

### 3.3 Physical form

**FIGURE 4:**
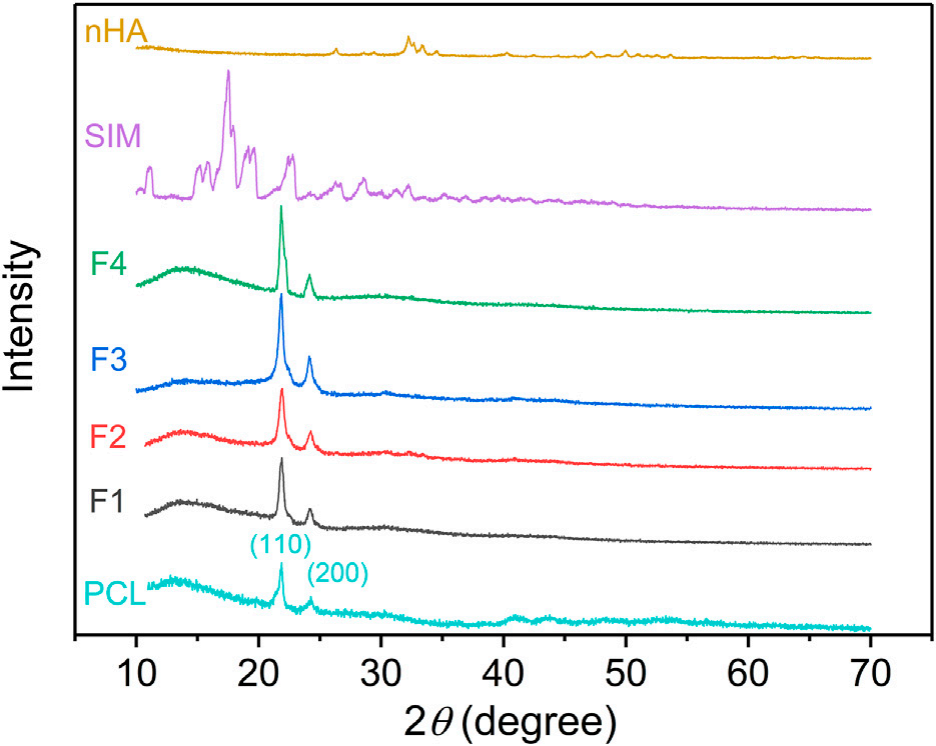
XRD pattern of the prepared nanofibers.

The physical form of powders (SIM and nHA) and the prepared nanofibers was exhibited in Figure 4. For PCL, the characteristic peaks at 21.8° and 24.2° were contributed to the crystal planes (110) and (200). The same peaks appeared on the XRD pattern of F1–F4 nanofibers suggested that PCL was the sole polymer (Chen et al., 2019; Longo et al., 2022). The characteristic peaks of PCL appeared on the XRD pattern of F1–F4 nanofibers, which was attributed to the unique polymer PCL. For nHA, the peaks at 26.4°, 32.2°, 33.4°, 34.5°, 40.3°, 47.1°, 50.0°, and 53.7° are contributed to the planes (002), (112), (300), (202), (310), (222), (213), and (004), respectively (El-Meliegy et al., 2018; Shafeeq et al., 2020). However, these characteristic peaks did not observe in the XRD pattern of F3 and F4 nanofibers. The low content of nHA was mostly located inside the nanofibers and cannot be detected. When the content of nHA reached 6%, nHA on the surface of nanofibers increased, thus, the obvious peaks of nHA appeared in the XRD pattern in Supplementary Figure S2. The addition of nHA did not change the crystal structure of PCL, and there was only weak hydrogen bond interaction with PCL. The XRD pattern of SIM exhibited several sharp peaks, indicating its crystalline structure, but F2 and F4 nanofibers did not have the relevant peaks of SIM. Despite increasing drug concentration in the nanofibers to 6%, the peaks of SIM were not observed in the XRD pattern, as shown in Supplementary Figure S2. During the electrospinning process in Figure 1B, the core working fluid was clear which means drugs and polymer were evenly distributed. The solvent in the spinning solution around the spinneret was evaporated at an extremely fast rate, the drug molecules had no time to aggregate and fixed into the solidified nanofibers. Extensive research has demonstrated that drug molecules could be transformed from crystalline to amorphous forms by electrospinning processes (Casian et al., 2019; Liu et al., 2023). This conclusion is further supported by the XRD analysis, where no characteristic peaks of SIM were observed in the XRD patterns of the drug-loaded fibers, regardless of the concentration of the drug.

### 3.4 Chemical compatibility of the fabricated nanofibers

**FIGURE 5:**
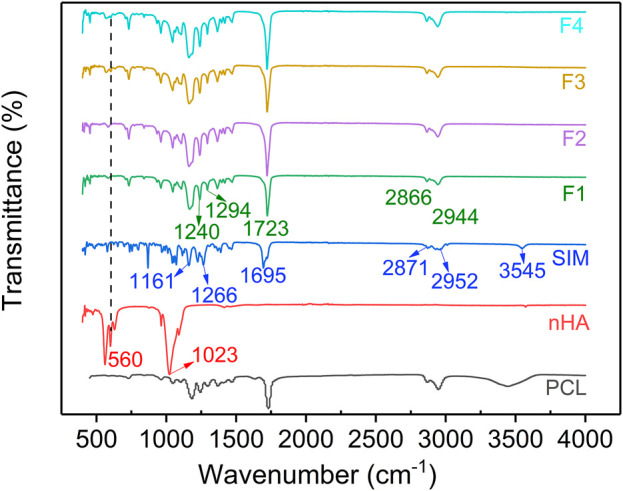
FTIR spectrum of the prepared nanofibers.

Figure 5 showed the FTIR spectrum of the prepared nanofibers. The FTIR spectrum of SIM had several peaks. The characteristic peaks at 1,161 cm^–1^ and 1,266 cm^–1^ were contributed to the C-O-C group, the peak at 1,695 cm^–1^ was contributed to the C=O group, the peaks at 2,871 cm^–1^ and 2,952 cm^–1^ were contributed to the stretching vibration of CH_2_ and CH_3_ groups, and the peak at 3,545 cm^–1^ was attributed to the stretching vibration of OH group (Malekpour et al., 2021; Delan et al., 2022; Pan et al., 2022). For nHA, the characteristic peaks at 560 cm^–1^ and 1,023 cm^–1^ contributed to the PO_4_^3–^ group (Cho et al., 2021; Azaryan et al., 2022; Moghadam Deymeh et al., 2022). In the FTIR spectrum of F1-F4, the peaks of PCL appeared at 2,944 cm^–1^、2,866 cm^–1^、1723 cm^–1^、1,294 cm^–1^, and 1,240 cm^–1^ (Rezk et al., 2019). The peaks of nHA at 560 cm^–1^ appeared in the IR spectrum of F3 and F4 nanofibers which indicated that nHA was successfully loaded into nanofibers. In addition, no correlative peaks observed in the IR spectrum of F2 and F4 nanofibers, which gave a hint of the good chemical compatibility between SIM and PCL. Recent studies have demonstrated that when SIM is loaded onto fibers by electrospinning, polymer and SIM can form hydrogen bonds through hydroxyl and carboxyl groups. This interaction can result in the disappearance of the characteristic peaks of SIM in the spectrum of the SIM-loaded fibers (Malekpour et al., 2021; Delan et al., 2022).

### 3.5 Thermodynamic stability

**FIGURE 6:**
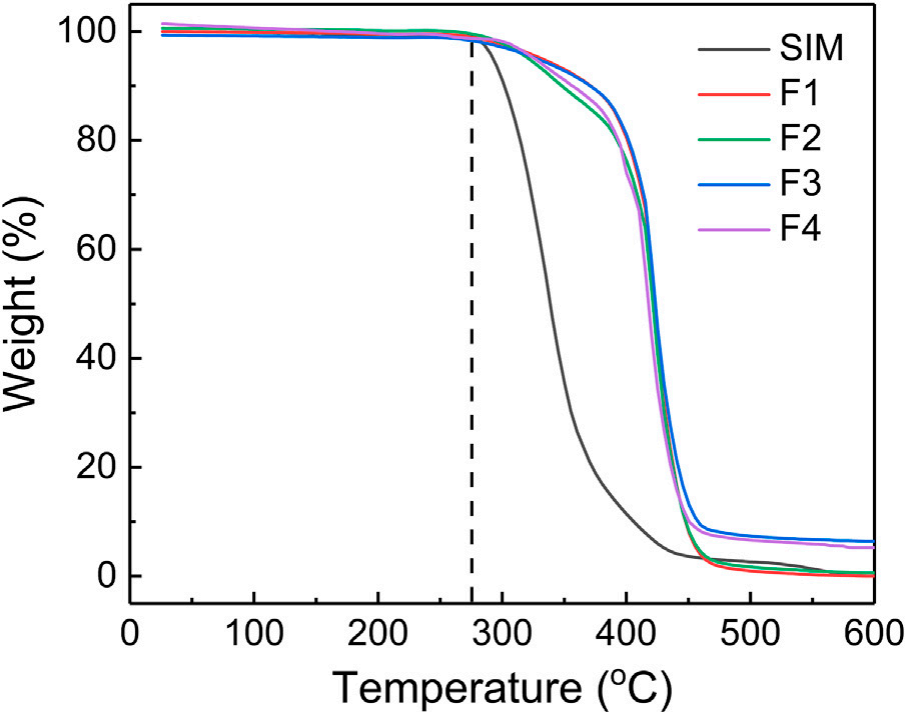
TGA of SIM and the prepared nanofibers.

TGA is a technique that measures the mass loss of materials as it is subjected to increasing temperatures. In this case, TGA was used to analyze the thermodynamic stability of electrospun nanofibers. SIM and PCL had same initial thermal decomposition temperature at 275°C, but the mass of SIM decreased faster than that of PCL in the next stage. In Figure 6, the TGA curve of F2 had a quicker decline than F1 after thermal decomposition, which can be attributed to the small amounts of drugs in F2. This also could explain the decline of F4 in the thermal decomposition stage. Comparing with the almost thermal decomposition of F1 and F2, F3, and F4 still had 6.45% and 5.26% residual mass, respectively. Theoretically, F3 and F4 have 4.76% and 3.45% nHA. Thus, the residual mass of F3 and F4 could be attributed to nHA within the allowable range of error.

### 3.6 *In vitro* drug release

**FIGURE 7:**
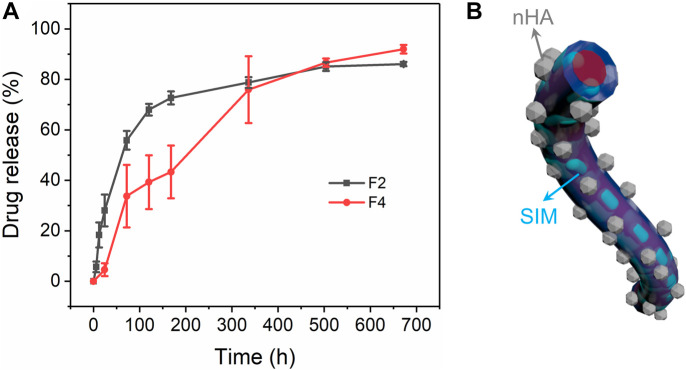
Drug release of monolithic F2 and core–sheath F4 nanofibers. **(A)** Drug release profiles of drug-loading nanofibers, **(B)** schematic diagram of core–sheath F4 nanofibers.

Monolithic F2 nanofibers and core–sheath F4 nanofibers show high drug-loading efficiency of 85.53% ± 3.76% and 82.12% ± 5.38%, respectively, indicating that the electrospinning process is effective for drug loading. In Figure 7A, monolithic F2 nanofibers exhibited a rapid drug release profile at the first 16 h, and stabilized in subsequent stage. This is attributed to the high specific surface area of electrospun nanofibers, which increases the contact area between the drug and water. In contrast, core–sheath F4 nanofibers had a sustained drug release profile throughout the entire 672 h. The sustained drug release process in the nanofiber can be approximated as the process of water intrusion. The sustained drug release behavior benefits from the core–sheath structure of F4 nanofibers, which prevents water molecules from invading the core and escaping the drugs from the core, as shown in Figure 7B. The drug release behavior from fibers was primarily influenced by the intrusion of water and the subsequent diffusion process. As shown in Supplementary Figure S1, the core–sheath F4 nanofiber membrane exhibited a narrower bandwidth compared to the other fibers, indicating a more tightly packed fiber structure. This tighter fiber membrane could potentially decrease the porosity of the fiber membrane, thereby hindering the penetration of water molecules. Additionally, the incorporation of nHA increased the roughness surface of fibers, resulting in a reduced contact area between water molecules and the fiber surface, and contributed to the delayed intrusion of water into the fibers to obtain the sustained release behavior.

Drug release behavior were further evaluated using several mathematical models in Table 2 (Hajiabbas et al., 2021). The high correlation coefficients in equations mean that both F2 and F4 nanofibers follow the first-order model. The order release of the drug was the optimal release behavior in all dosages. The higher *R* ^2^ in the zero-order equation of F4 nanofibers than that of F2 nanofibers indicated that F4 nanofibers had a relatively better zero-order drug release process than F2 nanofibers. Only the *R* ^2^ of F2 nanofibers exceeds 0.9 in the Higuchi equations, indicating that the drug dissolution in F4 nanofibers was mainly based on the porosity of the polymer. The higher *R* ^2^ in the Peppas equations for F2 and F4 nanofibers means that both nanofibers fit the Peppas model. In the Peppas model, *n* is the drug diffusion coefficient used to analyze the drug dissolution types. *n* in the Peppas equation of F2 nanofibers is less than 0.45 which suggests that the drug release in F2 nanofibers belongs to Fickian diffusion. For F4 nanofibers, *n* is 0.45 (between 0.45 and 0.9) which suggests that the drug release in F4 nanofibers has both Fickian diffusion and matrix erosion mechanisms. The Higuchi and Peppas models are only suitable for analyzing monolithic materials with uniform drug distribution. However, since the core-sheath structure of F4 nanofibers increases the complication of the drug dissolution process, the drugs were not evenly distributed in F4 nanofibers. Therefore, the Higuchi model and Peppas model may not be suitable for analyzing drug release behavior in core-sheath F4 nanofibers. Nonetheless, the core–sheath structure of nanofibers not only can improve the drug release behavior, but also has a positive impact on the dissolution mechanism of drugs.

**TABLE 2:** Fitting results of several mathematical models to drug release data.

No	First-order model	Zero-order model	Higuchi model	Rigter–Peppas model
F2	*y* = 82.37 [1-exp (−0.02*t)] (*R* ^2^ = 0.9918)	*y* = 28.03 + 0.11*t (*R* ^2^ = 0.5944)	*y* = 3.51 [*t*^(1/2)]+11.22 (*R* ^2^ = 0.8390)	*y* = 12.12*(*t*^0.32) (*R* ^2^ = 0.9114)
F4	*y* = 98.25 [1-exp (−0.01*t)] (*R* ^2^ = 0.9821)	*y* = 14.84 + 0.14*t (*R* ^2^ = 0.8612)	*y* = 3.94[*t*^(1/2)]-4.23 (*R* ^2^ = 0.9644)	*y* = 2.85*(*t*^0.54) (*R* ^2^ = 0.9623)

For fitting equations, *t* is the drug release time.

### 3.7 *In vitro* cell proliferation

**FIGURE 8:**
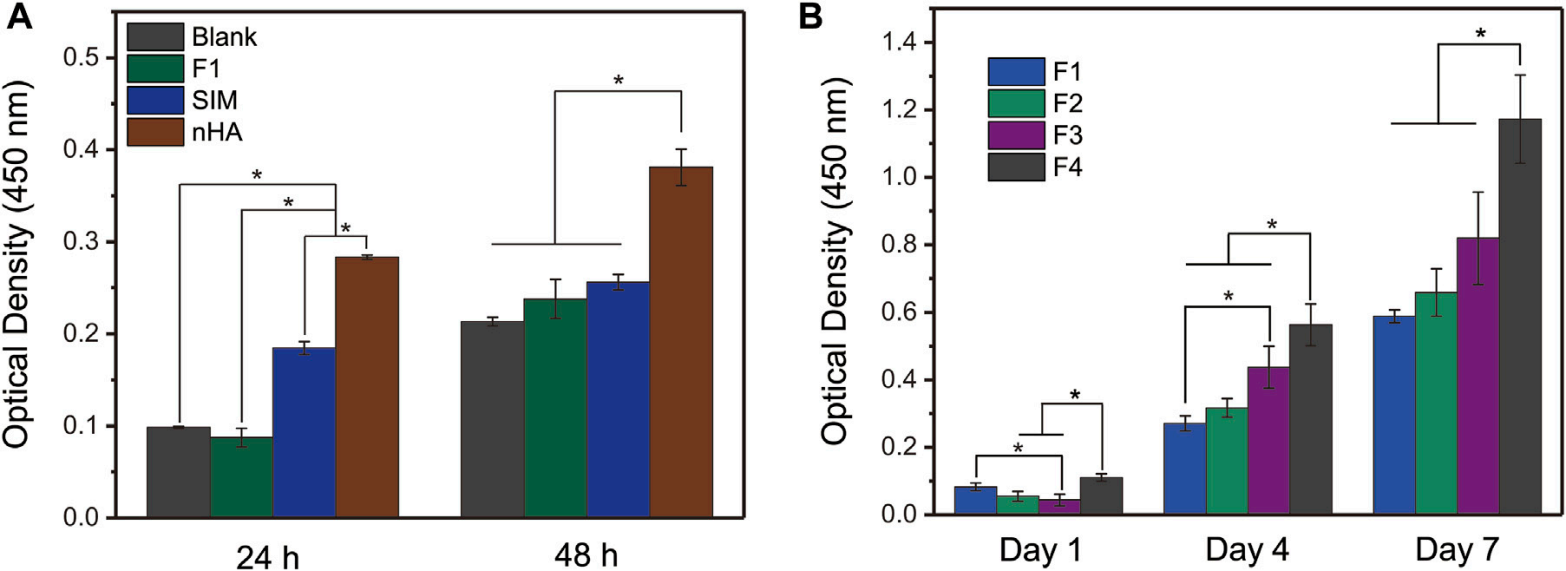
*In vitro* cell proliferation of **(A)** controlled samples and **(B)** prepared nanofibers. One-Way ANOVA Tukey’s test was conducted to explore the significant difference between nanofiber samples, * indicates *p* < 0.05.

As shown in Figure 8A, the pre-experiment was proposed to explore the cell proliferation of control samples within 48 h, including blank PBS group, SIM group, nHA group, and F1 fibers group. SIM exhibited a positive effect on cell proliferation, whereas F1 fibers composed of the only polymer PCL showed a good cytocompatibility. Compared with the blank group, F1 fibers did not show any inhibitory effects on cells. In this pre-experiment, nHA exhibited the best potential to promote cell proliferation than other groups. This pre-experiment has successfully demonstrated that the raw materials (including PCL, SIM, and nHA) are all beneficial to cell proliferation. It is interesting to investigate the potential of affordable bioactive substances in promoting bone regeneration, as an alternative to costly bone regenerative growth factors. The following study will focus on the cell proliferation properties of the prepared F1-F4 fibers which load these bioactive substances.

Figure 8B exhibited the cell proliferation of prepared nanofibers to evaluate their bioactivity. The nanofibers loaded with nHA or SIM had good cell proliferation behavior. In particular, the core–sheath nanofibers F4, which had a sustained drug release profile, had the best cell proliferation among the four nanofibers tested. This may be due to the core–sheath structure of F4 nanofibers, which allowed for the different storage locations of the bioactivity substances (nHA particles and SIM). The nHA particles on the sheath layer of F4 nanofibers could promote cell proliferation through physical contact, while the SIM stored inside the core layer of F4 nanofibers could promote cell proliferation in a sustained release manner. This synergistic effect on F4 nanofibers is not simply the sum of the effects of the individual components. Overall, the results suggest that the prepared nanofibers have good bioactivity and potential bone regeneration applications.

## 4 Conclusion

In this work, we successfully fabricated core–sheath nanofibers loaded with SIM and nHA simultaneously, resulting in nanofibers with a cylindrical morphology. The core–sheath structure and nHA nanoparticles were observed by TEM. The SIM existed in an amorphous form in nanofibers which had been confirmed by the XRD results, and the FTIR results indicated good chemical compatibility between the components of nanofibers. There was 3.45% nHA loaded in core–sheath nanofibers during the TGA analysis. The release time of SIM was controlled due to the protective effect of core–sheath structure, with a sustained release profile over 672 h. Cell proliferation tests showed that both SIM or nHA in nanofibers could promote cell proliferation, with the optimal behavior exhibited by the core–sheath F4 nanofibers loaded with both nHA and sustained-release SIM. The synergistic effect of the nanostructures and materials used in this study suggests the potential for promoting bone regeneration and may inspire further research in the development of other biomedical materials with similar synergistic effects.

## Data Availability

The raw data supporting the conclusion of this article will be made available by the authors, without undue reservation.
