# Supplementary material for: Electrospun core–sheath PCL nanofibers loaded with nHA and simvastatin and their potential bone regeneration applications
Source: Front Bioeng Biotechnol. 2023 Jul 26;11:1205252. doi: 10.3389/fbioe.2023.1205252 (PMC10410860; doi:10.3389/fbioe.2023.1205252)
Supplement: Supplementary file 1 [file DataSheet1.docx]

Supplementary Material

Electrospun core–sheath PCL nanofibers loaded with nHA and simvastatin and their potential bone regeneration applications

Chenghui Qian^a, b, †^, Yubo Liu^c, †^, Si Chen^a, b^, Chenyang Zhang^a, b^, Xiaohong Chen^c, d^, Yuehua Liu^a, b, *^, Ping Liu^c, d, *^

^a^Shanghai Stomatological Hospital & School of Stomatology, Fudan University, No.356 East Beijing Road, Shanghai, China.

^b^Shanghai Key Laboratory of Craniomaxillofacial Development and Diseases, Fudan University

^c^School of Materials and Chemistry, University of Shanghai for Science & Technology, Shanghai 200093, China

^d^Shanghai Engineering Technology Research Center for High-Performance Medical Device Materials, Shanghai 200093, China

^†^Chenghui Qian and Yubo Liu contributed equally to this work

*** Correspondence:** Yuehua Liu: [liuyuehua@fudan.edu.cn](mailto:liuyuehua@fudan.edu.cn); Ping Liu: [pingliu_1962@163.com](mailto:pingliu_1962@163.com)


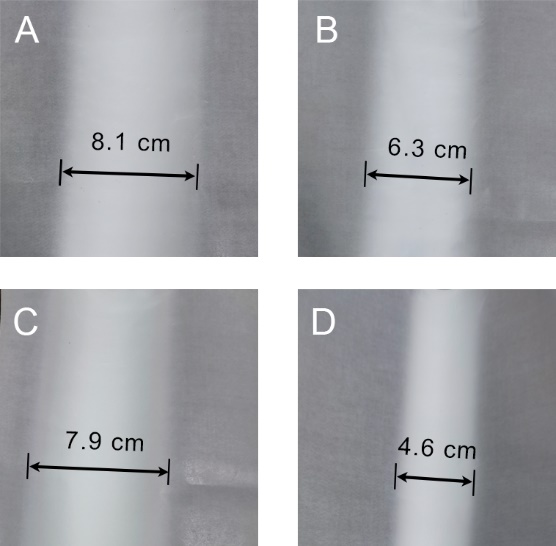


**Supplementary Figure S1.** The fiber membrane bandwidth of prepared F1-F4 fibers, (A) F1 fibers, (B) F2 fibers, (C) F3 fibers, and (D) F4 fibers.





**Supplementary Figure S2.** XRD pattern of fibers with increased nHA and SIM content. The PCL fibers containing 6% SIM was defined as F5, and the PCL fibers containing 6% nHA was defined as F6.
